# Complete Genome Sequence of Cronobacter dublinensis Bacteriophage vB_Cdu_VP8

**DOI:** 10.1128/mra.00296-23

**Published:** 2023-05-17

**Authors:** Michal Kajsik, Peter Durovka, Maria Kajsikova, Miroslav Bohmer, Tomas Szemes, Hana Drahovska

**Affiliations:** a Medirex Group Academy n.o., Nitra, Slovakia; b Comenius University Science Park, Bratislava, Slovakia; c Institute of Molecular Biology, Slovak Academy of Sciences, Bratislava, Slovakia; Queens College Department of Biology

## Abstract

Cronobacter dublinensis is a Gram-negative pathogen that is capable of causing infection in humans. In this announcement, we describe the characterization of bacteriophage vB_Cdu_VP8, which is able to lyse a Cronobacter dublinensis strain. Related to phages belonging to the genus *Muldoonvirus*, such as Muldoon and SP1, vB_Cdu_VP8 contains 264 predicted protein-coding genes and 3 tRNAs.

## ANNOUNCEMENT

The genus *Cronobacter* consists of opportunistic pathogens that have the ability to cause infections in newborns, such as meningitis, necrotizing enterocolitis, and sepsis (1). *Cronobacter* spp. are also capable of causing infections in elderly individuals and immunocompromised adults (2). Infections caused by Cronobacter dublinensis appear to be less common than those caused by other species of the genus *Cronobacter*; however, there is evidence to suggest that this pathogen can cause serious infections in humans (3, 4). This announcement describes the process of isolating, sequencing, and annotating the genome of the bacteriophage vB_Cdu_VP8. The phage was obtained by growth on Cronobacter dublinensis strain 582 (5), which was cultivated in LB broth with filtered wastewater (0.22-μm pore size) at 37°C. The wastewater sample was collected in September 2019 from a wastewater treatment plant in Bratislava, Slovakia. The host specificity was determined using the double agar overlay plaque assay (6). The phage was then purified through repeated isolations from single plaques on double agar and amplified in liquid cultures. Further purification was achieved by subjecting the phage to ultracentrifugation in a cesium chloride gradient (7). Bacteriophage DNA was purified using a phage DNA isolation kit (NorgenBiotek, Canada). Next-generation sequencing libraries were prepared with a Nextera XT DNA library preparation kit, and phage vB_Cdu_VP8 was sequenced on an Illumina MiSeq platform (paired-end 150-bp reads) using 300-cycle v2 chemistry. FastQC (www.bioinformatics.babraham.ac.uk/projects/fastqc) was used to control the quality of the 185,958 sequence reads, which were subsequently trimmed using Trimmomatic v0.39 and assembled using SPAdes v3.5.0 (8, 9). The assembly resulted in a single contig with 277× coverage. To finalize the sequence and ensure that the complete termini would be present, PCR products (forward, 5′-GGCAGATTCGACGCTAAAGG-3′; reverse, 5′-TTGTTGAAACCTACTGCGCC-3′) amplified from the contig ends were Sanger sequenced. Protein-coding genes were predicted using GLIMMER v3.0, and tRNA coding sequences were localized using ARAGORN v1.2.41 (10, 11). The genomic terminus type was predicted with PhageTerm (12). Functional annotations were guided by results from RASTtk v2.0, MetaGeneAnnotator, and BLAST v2.2.31 searches against the NCBI nonredundant and Swiss-Prot databases (13–15). All analyses were conducted with default settings. Whole-genome comparisons were performed with the Average Nucleotide Identity (ANI) Calculator (https://www.ezbiocloud.net/tools/ani) using the OrthoANIu algorithm (16). The phage life cycle was predicted with the PhageAI tool (17).

Bacteriophage vB_Cdu_VP8 was predicted to be virulent, with an estimated accuracy of 97.56%. It is able to infect and lyse Cronobacter dublinensis strain 582 (NCTC 9844) (5). vB_Cdu_VP8 has a 166,625-bp genome with a G+C content of 41.89%. A total of 264 coding sequences and 3 tRNA genes (tRNA-Met-CAT, tRNA-Arg-TCT, and tRNA-Gly-TTC) were annotated, resulting in a protein-coding density of 95.2%. Phage vB_Cdu_VP8 shares high nucleotide-level similarity with phages from the class *Caudoviricetes* and the genus *Muldoonvirus*, such as *Serratia* phages Muldoon (GenBank accession number NC_049464) (18), SP1 (GenBank accession number OM457001) (19), and PS2 (GenBank accession number KJ025957) (20), with ANI values of 95.94%, 92.66%, and 84.78%, respectively.

### Data availability.

The genome sequence of bacteriophage vB_Cdu_VP8 was deposited under GenBank accession number OQ361773, BioProject accession number PRJNA922742, SRA accession number SRR23097098, and BioSample accession number SAMN32709064.

## References

[1] Hunter CJ, Bean JF. 2013. *Cronobacter*: an emerging opportunistic pathogen associated with neonatal meningitis, sepsis and necrotizing enterocolitis. J Perinatol 33:581–585. doi:10.1038/jp.2013.26. 23538645

[2] Alsonosi A, Hariri S, Kajsik M, Orieskova M, Hanulik V, Roderova M, Petrzelova J, Kollarova H, Drahovska H, Forsythe S, Holy O. 2015. The speciation and genotyping of *Cronobacter* isolates from hospitalised patients. Eur J Clin Microbiol Infect Dis 34:1979–1988. doi:10.1007/s10096-015-2440-8. 26173692 PMC4565866

[3] Cruz-Córdova A, Rocha-Ramírez LM, Ochoa SA, González-Pedrajo B, Espinosa N, Eslava C, Hernández-Chiñas U, Mendoza-Hernández G, Rodríguez-Leviz A, Valencia-Mayoral P, Sadowinski-Pine S, Hernández-Castro R, Estrada-García I, Muñoz-Hernández O, Rosas I, Xicohtencatl-Cortes J. 2012. Flagella from five *Cronobacter* species induce pro-inflammatory cytokines in macrophage derivatives from human monocytes. PLoS One 7:e52091. doi:10.1371/journal.pone.0052091. 23284883 PMC3528739

[4] Cruz A, Xicohtencatl-Cortes J, González-Pedrajo B, Bobadilla M, Eslava C, Rosas I. 2011. Virulence traits in *Cronobacter* species isolated from different sources. Can J Microbiol 57:735–744. doi:10.1139/w11-063. 21859256

[5] Joseph S, Desai P, Ji Y, Cummings CA, Shih R, Degoricija L, Rico A, Brzoska P, Hamby SE, Masood N, Hariri S, Sonbol H, Chuzhanova N, McClelland M, Furtado MR, Forsythe SJ. 2012. Comparative analysis of genome sequences covering the seven *Cronobacter* species. PLoS One 7:e49455. doi:10.1371/journal.pone.0049455. 23166675 PMC3500316

[6] Kropinski AM, Mazzocco A, Waddell TE, Lingohr E, Johnson RP. 2009. Enumeration of bacteriophages by double agar overlay plaque assay. Methods Mol Biol 501:69–76. doi:10.1007/978-1-60327-164-6_7. 19066811

[7] Boulanger P. 2009. Purification of bacteriophages and SDS-PAGE analysis of phage structural proteins from ghost particles. Methods Mol Biol 502:227–238. doi:10.1007/978-1-60327-565-1_13. 19082559

[8] Bolger AM, Lohse M, Usadel B. 2014. Trimmomatic: a flexible trimmer for Illumina sequence data. Bioinformatics 30:2114–2120. doi:10.1093/bioinformatics/btu170. 24695404 PMC4103590

[9] Bankevich A, Nurk S, Antipov D, Gurevich AA, Dvorkin M, Kulikov AS, Lesin VM, Nikolenko SI, Pham S, Prjibelski AD, Pyshkin AV, Sirotkin AV, Vyahhi N, Tesler G, Alekseyev MA, Pevzner PA. 2012. SPAdes: a new genome assembly algorithm and its applications to single-cell sequencing. J Comput Biol 19:455–477. doi:10.1089/cmb.2012.0021. 22506599 PMC3342519

[10] Delcher AL, Bratke KA, Powers EC, Salzberg SL. 2007. Identifying bacterial genes and endosymbiont DNA with Glimmer. Bioinformatics 23:673–679. doi:10.1093/bioinformatics/btm009. 17237039 PMC2387122

[11] Laslett D, Canback B. 2004. ARAGORN, a program to detect tRNA genes and tmRNA genes in nucleotide sequences. Nucleic Acids Res 32:11–16. doi:10.1093/nar/gkh152. 14704338 PMC373265

[12] Garneau JR, Depardieu F, Fortier L-C, Bikard D, Monot M. 2017. PhageTerm: a tool for fast and accurate determination of phage termini and packaging mechanism using next-generation sequencing data. Sci Rep 7:8292. doi:10.1038/s41598-017-07910-5. 28811656 PMC5557969

[13] Noguchi H, Taniguchi T, Itoh T. 2008. MetaGeneAnnotator: detecting species-specific patterns of ribosomal binding site for precise gene prediction in anonymous prokaryotic and phage genomes. DNA Res 15:387–396. doi:10.1093/dnares/dsn027. 18940874 PMC2608843

[14] Camacho C, Coulouris G, Avagyan V, Ma N, Papadopoulos J, Bealer K, Madden TL. 2009. BLAST+: architecture and applications. BMC Bioinformatics 10:421. doi:10.1186/1471-2105-10-421. 20003500 PMC2803857

[15] UniProt Consortium. 2019. UniProt: a worldwide hub of protein knowledge. Nucleic Acids Res 47:D506–D515. doi:10.1093/nar/gky1049. 30395287 PMC6323992

[16] Yoon S-H, Ha S-M, Lim J, Kwon S, Chun J. 2017. A large-scale evaluation of algorithms to calculate average nucleotide identity. Antonie Van Leeuwenhoek 110:1281–1286. doi:10.1007/s10482-017-0844-4. 28204908

[17] Tynecki P, Guziński A, Kazimierczak J, Jadczuk M, Dastych J, Onisko A. 2020. PhageAI: bacteriophage life cycle recognition with machine learning and natural language processing. bioRxiv. 2020.07.11.198606. doi:10.1101/2020.07.11.198606.

[18] Campbell S, Atkison C, Moreland R, Liu M, Ramsey J, Leavitt J. 2020. Complete genome sequence of phage Muldoon. Microbiol Resour Announc 9:e01418-19. doi:10.1128/MRA.01418-19. 31896655 PMC6940307

[19] Tikhe CV, Dimopoulos G. 2022. Phage therapy for mosquito larval control: a proof-of-principle study. mBio 13:e03017-22. doi:10.1128/mbio.03017-22. 36445694 PMC9765668

[20] Teng T, Zhang G, Fan X, Zhang Z, Zhang L, Wu D, Chen S, Li Y, Jin J. 2018. Complete genome sequence analysis of PS2, a novel T4-like bacteriophage that infects *Serratia marcescens* clinical isolates. Arch Virol 163:1997–2000. doi:10.1007/s00705-018-3803-0. 29574589

